# Oncolytic vaccinia virus GLV-1h68 strain shows enhanced replication in human breast cancer stem-like cells in comparison to breast cancer cells

**DOI:** 10.1186/1479-5876-10-167

**Published:** 2012-08-17

**Authors:** Huiqiang Wang, Nanhai G Chen, Boris R Minev, Aladar A Szalay

**Affiliations:** 1 Institute of Biochemistry, Biocenter, University of Würzburg, Am hubland, D-97074, Würzburg, Germany; 2 Genelux Corporation, San Diego Science Center, 3030 Bunker Hill Street, Suite 310, San Diego, CA, 92109, USA; 3 Department of Radiation Oncology, Rebecca & John Moores Comprehensive Cancer Center, University of California, San Diego, La Jolla, CA, 92093, USA; 4 UCSD Division of Neurosurgery, University of California, San Diego, La Jolla, CA, 92093, USA; 5 Rudolf Virchow Center for Experimental Biomedicine and Institute for Molecular Infection Biology, University of Würzburg, Am hubland, D-97074, Würzburg, Germany

**Keywords:** Aldehyde dehydrogenase 1, Cancer stem cells, Oncolytic virotherapy, Vaccinia virus, EMT

## Abstract

**Background:**

Recent data suggest that cancer stem cells (CSCs) play an important role in cancer, as these cells possess enhanced tumor-forming capabilities and are responsible for relapses after apparently curative therapies have been undertaken. Hence, novel cancer therapies will be needed to test for both tumor regression and CSC targeting. The use of oncolytic vaccinia virus (VACV) represents an attractive anti-tumor approach and is currently under evaluation in clinical trials. The purpose of this study was to demonstrate whether VACV does kill CSCs that are resistant to irradiation and chemotherapy.

**Methods:**

Cancer stem-like cells were identified and separated from the human breast cancer cell line GI-101A by virtue of increased aldehyde dehydrogenase 1 (ALDH1) activity as assessed by the ALDEFLUOR assay and cancer stem cell-like features such as chemo-resistance, irradiation-resistance and tumor-initiating were confirmed in cell culture and in animal models. VACV treatments were applied to both ALDEFLUOR-positive cells in cell culture and in xenograft tumors derived from these cells. Moreover, we identified and isolated CD44^+^CD24^+^ESA^+^ cells from GI-101A upon an epithelial-mesenchymal transition (EMT). These cells were similarly characterized both in cell culture and in animal models.

**Results:**

We demonstrated for the first time that the oncolytic VACV GLV-1h68 strain replicated more efficiently in cells with higher ALDH1 activity that possessed stem cell-like features than in cells with lower ALDH1 activity. GLV-1h68 selectively colonized and eventually eradicated xenograft tumors originating from cells with higher ALDH1 activity. Furthermore, GLV-1h68 also showed preferential replication in CD44^+^CD24^+^ESA^+^ cells derived from GI-101A upon an EMT induction as well as in xenograft tumors originating from these cells that were more tumorigenic than CD44^+^CD24^-^ESA^+^ cells.

**Conclusions:**

Taken together, our findings indicate that GLV-1h68 efficiently replicates and kills cancer stem-like cells. Thus, GLV-1h68 may become a promising agent for eradicating both primary and metastatic tumors, especially tumors harboring cancer stem-like cells that are resistant to chemo and/or radiotherapy and may be responsible for recurrence of tumors.

## Background

There is an increasing body of knowledge that human breast cancers are driven by a tumor-initiating “cancer stem cells” (CSCs) component that may contribute to tumor metastases and therapeutic resistance [1-5]. Breast CSCs were initially characterized as CD44^+^/CD24^−^/lin^−^ cells that were capable of serial transplantation in non-obese/severe combined immunodeficient (NOD/SCID) mice [6]. In addition to these markers, Ginestier *et.al* have recently shown that cells with stem cell properties in both normal and malignant breast samples can be identified by the expression of the enzyme aldehyde dehydrogenase 1 (ALDH1). By using flow cytometry and the ALDEFLUOR assay that measures ALDH1 activity, CSCs were isolated from primary human mammary carcinomas grown as xenografts in NOD/SCID mice. In addition, ALDH1 immunostaining identified both normal and malignant stem cells *in situ* in fixed paraffin embedded sections [7]. Furthermore, recent data suggests that immortalized cell lines derived from both murine and human tissues may also contain a cellular population displaying stem cell properties [8-11]. By analyzing thirty-three breast cancer cell lines, Charafe-Jauffret *et al.* confirmed the hierarchical organization of immortalized cell lines and identified ALDH1 as a potential stem cell marker and therapeutic target [12].

Recently, the involvement of an Epithelial-Mesenchymal Transition (EMT) in the metastatic dissemination of epithelial cancer cells has emerged in cancer biology as a novel concept. Using a mammary tumor progression model, it was shown that cells possessing both stem and tumorigenic characteristics of CSCs can be derived from human mammary epithelial cells following the activation of the Ras-MAPK pathway and that the acquisition of stem and tumorigenic characters is driven by EMT induction [13,14]. Moreover, the EMT cell model has successfully been utilized for screening for agents with mammary epithelial CSCs-specific toxicity [15].

Advances in cancer research has resulted in increased detection, improved treatments and enhanced prevention of metastases. Despite these advances, however, metastatic cancers are generally resistant to conventional therapeutics and the prognosis is poor. Therefore, there is an urgent need for the development of new therapies and novel approaches which target cancer metastases. A growing body of scientific evidence indicates that oncolytic vaccinia viruses (VACVs) carrying imaging genes may represent a new treatment strategy that combines tumor site-specific therapeutics with diagnostics (theranostics) [16].

The human breast cancer cell line GI-101A was widely used as a model for testing oncolytic VACV theranostics in our groups [17-24]. To demonstrate the efficacy of vaccinia virotherapy against cancer stem-like cells, we isolated ALDEFLUOR-positive and ALDEFLUOR-negative cells from GI-101A cells and applied the viral treatment to these cells. The evidence from cell culture and tumor xenograft studies indicated that GI-101A-derived ALDEFLUOR-positive cells possessed CSC properties. Compared to ALDEFLUOR-negative cells, the oncolytic VACV GLV-1h68 strain showed enhanced replication in the ALDEFLUOR-positive cells and was also able to eradicate the xenograft tumors derived from ALDEFLUOR-positive cells. Moreover, we isolated CD44^+^CD24^+^ESA^+^ and CD44^+^CD24^-^ESA^+^ cells from GI-101A cell line upon an EMT induction. The CD44^+^CD24^+^ESA^+^ cells demonstrated increased tumorigenicity than CD44^+^CD24^-^ESA^+^ cells. Interestingly, GLV-1h68 strain showed enhanced replication in CD44^+^CD24^+^ESA^+^ cells in contrast to CD44^+^CD24^-^ESA^+^ cells and better therapeutic efficacy in the xenograft tumors derived from the CD44^+^CD24^+^ESA^+^cells.

## Methods

### Cell culture

Human breast cancer cell lines (MCF-7, MDA-MB-231, and HS578T) and African green monkey kidney fibroblasts (CV-1) were obtained from the American Type Culture Collection (ATCC). SUM149PT was purchased from Asterand, PLC (Detroit, MI). The cell line GI-101A, a highly metastatic derivative of GI-101 human ductal adenocarcinoma [25-27], was kindly provided by Dr. A. Aller (Rumbaugh-Goodwin Institute for Cancer Research, Inc.). The cell lines were grown under the recommended culture conditions (Additional file 1: Table S1, Additional file 2: Figure S2). All experiments were done with semi-confluent cells in the exponential phase of growth. Immortalized human mammary epithelial cells (HMLE) were kindly provided and cultured as recommended by Dr. R.A. Weinberg (Whitehead Institute, Cambridge, MA). HMLE were cultured in 1:1 Dulbecco’s Modified Eagle’s Medium (DMEM)/F12 medium (Cellgro) complemented with 10% FBS (Cellgro), 100 U/ml penicillin-streptomycin (Cellgro), 2 mM/L glutamine (Cellgro), 10 ng/ml human epidermal growth factor (EGF) (Stemgent), 0.5 mg/ml hydrocortisone (Lonza) and 10 mg/ml insulin (Sigma). EMT was induced with additional 10 ng/ml recombinant human transforming growth factor (TGF-β1) (Stemgent) for 12 days. Similar culture condition was utilized to induce EMT in GI-101A cells.

### ALDEFLUOR assay and isolation of the ALDEFLUOR-positive cell population by fluorescence-activated cell sorting

ALDH1 activity was assessed in five human breast cancer cell lines using the ALDEFLUOR kit (StemCell Technologies). The population with higher ALDH1 enzymatic activity was isolated using a FACSAria™ III (Becton Dickinson), as previously described [7], and analyzed using Cell Lab Quanta™ SC MPL (Beckman Coulter). Briefly, 1 **×** 10^6^ cells were incubated in 1 ml ALDEFLUOR assay buffer containing ALDH1 substrate (1 **×** 10^-6^ M). In each experiment, a sample of cells was stained under identical conditions in the presence of 50 mM diethylaminobenzaldehyde, a specific ALDH1 inhibitor, as a negative control. The electronic sorting gates were set to exclude dead cells, doublets and aggregates. After sorting, the purity of sorted populations was examined using a double sorting of 10,000 ALDEFLUOR-positive and ALDEFLUOR-negative cells. The sorted ALDEFLUOR-positive population contained >80% of ALDEFLUOR-positive cells. On contrast, no ALDEFLUOR-positive cells were detected in the ALDEFLUOR-negative population.

### EMT induction assay and isolation of the CD44^+^CD24^+^ESA^+^ population by fluorescence-activated cell sorting

For EMT induction, HMLE and GI-101A cell lines were treated with EGF and TGF-β1 as described previously. The immunostaining was performed using the following antibodies against EMT markers: anti-E-cadherin (clone 36) (BD Transduction Laboratories), anti-Vimentin (clone V9) (Sigma) and anti-Fibronectin (clone 10) (BD Transduction Laboratories). Identification and sorting of CD44^+^CD24^+^ESA^+^ and CD44^+^CD24^-^ESA^+^ were performed using anti-human monoclonal antibodies such as anti-CD44-APC (clone G44-26) (BD Pharmingen), anti-CD24-PE (clone ML5) (BD Pharmingen) and anti-ESA-FITC (clone VU-1D9) (StemCell Technologies) on a BD FACSAria™ III cell sorter as described by the manufacturer.

### Flow cytometry

Nonconfluent cell cultures were trypsinized and the cell concentration was determined. Then the single cell suspension was washed with 1 **×** DPBS (Mediatech), and stained with antibodies recognizing human cell surface markers such as anti-CD44-APC (clone G44-26) (BD Pharmingen), anti-CD24-FITC (clone ML5) (BD Pharmingen), and anti-CD49f-PE (clone GoH3) (BD Pharmingen). Approximately 10,000 cells were incubated with antibodies for 30 minutes at room temperature according to manufacturer’s instructions. The unbound antibodies were washed off and cells were analyzed within 1 hour after staining on a BD FACSAria™ III (Becton Dickinson). All flow cytometry data were analyzed by Flowjo software (Treestar).

### Animal models and testing for tumorigenicity

Tumorigenicity of GI-101A ALDEFLUOR-positive and ALDEFLUOR-negative cells or CD44^+^CD24^+^ESA^+^ and CD44^+^CD24^-^ESA^+^ cells were assessed by measuring the efficiency of tumor formation in the abdominal mammary gland fat pad of 6–8 weeks old athymic nude mice (NCI/Hsd/Athymic Nude-*Foxn1*^nu^, Harlan). After sorting, the limiting dilutions (50,000, 5,000, and 500 cells) of the ALDEFLUOR-positive and ALDEFLUOR-negative population or 10,000 and 1,000 cells of CD44^+^CD24^+^ESA^+^ and CD44^+^CD24^-^ESA^+^ population of GI-101A cells were mixed with Matrigel (BD Biosciences; 1:1) and implanted to the mammary fat pads. Tumor growth was recorded weekly in two dimensions using a digital caliper. Tumor volume was calculated as [(length **×** width **×** width)/2] and reported in cubic millimeter. All animal experiments were conducted in accordance with accepted standards of humane animal care and all experiments were approved by the Animal Care and Use Committee at Explora Biolabs (San Diego Science Center).

### Virotherapy of GI-101A ALDEFLUOR or CD44^+^CD24^+^ESA^+^ xenograft tumors with GLV-1h68 strain

To assess the response of GI-101A tumors derived from ALDEFLUOR-positive and ALDEFLUOR-negative cells and derived from CD44^+^CD24^+^ESA^+^ and CD44^+^CD24^-^ESA^+^ cells, tumor colonization experiments with retro-orbitally injected GLV-1h68 strain in nude mice were carried out. 12 weeks after implantation of 5,000 ALDEFLUOR sorted cells or 22 weeks after implantation of 10,000 marker-sorted cells, a single dose of GLV-1h68 strains (5 **×** 10^6^ pfu in 100 μL 1 **×** DPBS) was retro-orbitally injected into tumor-bearing mice. A total of 30 mice, 5 for each dose group, were included in this study. Tumor measurements were recorded weekly for 7 weeks after initial virus injection, in parallel with fluorescence imaging of tumors.

### Anchorage-independent culture of tumor cells

ALDEFLUOR-positive and ALDELFUOR-negative cells from GI-101A were plated as single cells in 96-well ultra-low attachment plates (Corning) at different densities (1, 10, and 100 viable cells/well). Cells were grown in MammoCult^®^ human medium Kit (StemCell Technologies) for 12 days as described [28]. The capacity of cells to form mammospheres was quantified using ImageJ software (NIH, USA).

### Cytotoxicity of drugs in cell cultures

Fluorouracil (5-FU), *cis*-Diammine (1, 1-cyclobutanedicarboxylato) platinum (II) (Carboplatin), *cis*-diamminedichloroplatinum (II) (CDDP) (Cisplatin), Mitomycin C and Salinomycin were purchased from Sigma Aldrich; 3-(4, 5-Dimethylthiazol-2-yl)-2, 5-diphenyltetrazolium bromide (MTT) cellular proliferation assays were used for quantitation of chemo-cytotoxicity as described [18]. ALDEFLUOR-positive and ALDELFUOR-negative cells (50,000) from GI-101A were plated in 96-well plates in growth medium overnight and, thereafter, in serum-free medium. Cells were exposed to increasing concentrations (10^-9^, 10^-8^, 10^-7^, 10^-6^, 10^-5^, 10^-4^ M) of drugs (diluted in DMSO) for 96 hours, followed by the removal of media and the addition of 500 μL MTT (2.5 mg/mL) in RMPI without phenol red, for further culturing at 37°C and 5% CO_2_ for 4 hours . MTT solution was then removed and 400 μL isopropanol containing 1 N HCl was added. The samples (3 **×** 100 μL) were transferred to a 96-well plate and the absorbance was determined at 570 nm, as well as 650 nm in a SpectraMax M5 plate reader (Molecular Devices). Cell survival was calculated using the following formula: % cell survival = (absorbance value of treated cells/absorbance value of untreated control cells) **×** 100%.

### Tumor cell sensitivity to irradiation

For clonogenic assays, ALDEFLUOR-positive and ALDELFUOR-negative cells (10^5^ cells/mL) from GI-101A were irradiated at room temperature with a RS 2000 X-ray Biological Irradiator (Rad Source Technologies, Inc) at a dose rate of 4.95 Gy/minute. A dose curve of 0, 0.5, 1, 2, and 4 Gy was also generated. All controls were sham irradiated. To determine cell survival, colony forming assays were performed immediately after irradiation by plating cells in triplicate into 6-well plates. After a growth period of 28 days, cells were fixed with 75% ethanol and stained with 1% crystal violet, and colonies containing more than 50 cells were counted.

### Cell invasion assays

Invasion assays were done using Cultrex^®^ 96-well Basement Membrane Extract (BME) Cell Invasion Assay (Trevigen). Briefly, triplicate transwell chambers with 8-μm pore polycarbonate filters were coated with 50 μL of ice-cold 0.5× BME in DMEM/F12 and incubated for one hour at 37°C. Then cells in 200 μL of serum-free medium were added to the upper chamber. To monitor cell migration, 5,000 cells were seeded on the BME-coated filters and the lower chamber was filled with 600 μL of medium without or supplemented with 10% fetal bovine serum (Cellgro). After incubation for 48 hours, the cells on the underside of the filter were quantified by using Calcein-AM according to the assay kit manual.

### Viral replication in cell cultures

Sorted ALDEFLUOR-positive and ALDELFUOR-negative GI-101A cells (1 **×** 10^5^) or CD44^+^CD24^+^ESA^+^ and CD44^+^CD24^-^ESA^+^ cells (1 **×** 10^5^) were seeded into 24-well plates in triplicate and were infected with GLV-1h68 strain at an MOI of 10 or 0.01. The cells were incubated at 37°C for 1 hour with brief agitation every 10 minutes to aid infection. Then the infection medium was removed and cells were incubated in fresh growth medium until cell harvest at 1, 12, 24, 48, 72 hours post infection. Viral particles from the infected cells were released by performing a quick freeze-thaw cycle and the titers determined by plaque assays in CV-1 cell monolayers and recorded as plaque forming units (pfu)/million cells in duplicate. For the infection of EMT cells, HMLE and GI-101A cells were infected with GLV-1h68 or GLV-1h190 strain at an MOI of 10 after 12 day EMT induction. After incubation at 37°C for 1 hour, the infection medium was removed and replaced with fresh growth medium. Virus infection was monitored at different time points by taking images of same cells under a fluorescence microscope.

### Statistical analysis

Results are presented as the mean **±** standard deviation, calculated from a minimum of three repeated individual experiments for each group. Statistical analyses were done with SPSS (version 10, SPSS, Inc.). The comparisons among treatment groups were made using ANOVA, and the differences between groups were analyzed using LSD test when the ANOVA showed an overall significance. Statistical analysis of survival time was assessed using the log-rank test. P values of <0.05 were considered significant.

## Results

### Isolation and tumorigenicity of ALDEFLUOR-positive, cancer stem-like cells from GI-101A breast cancer cell line

**Figure 1:**
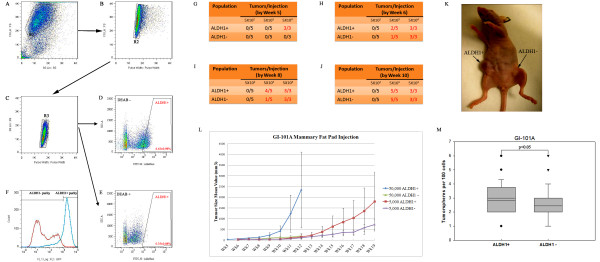
**ALDEFLUOR-positive cell population from GI-101A cells displays properties of CSCs in both cell culture and animal models.** (**A**-**F**) Representative FACS sorting of ALDEFLUOR-positive cells from GI-101A cell line. The ALDEFLUOR sorting on GI-101A cells was electronically gated firstly to exclude dead cells (R1), doublets (R2) and aggregates (R3). Incubation of cells with ALDEFLUOR substrate in the absence of DEAB induces a shift in BAAA fluorescence, defining the ALDEFLUOR-positive population, which represents 6.43 **±** 0.98% of the total live single cells in GI-101A comparing 0.35 **±** 0.98% of the total live single cells in the presence of DEAB. The purity of sorted cells was checked finally. (**G**-**L**) The ALDEFLUOR-positive cells have higher tumorigenic potential and latency in athymic nude mice. Tumor-forming occurrence was observed at different time points (G-J). (K) Representative tumor development in nude mouse at the ALDEFLUOR-positive cells’ injection site (50,000 cells injected). Smaller tumor was detected at the ALDEFLUOR-negative cells’ injection site (50,000 cells injected). (L) Tumor growth was monitored weekly by measuring the tumor volume of five mice in each group. The curves were plotted for the numbers of cells injected (50,000 cells and 5,000 cells) and for each population (ALDEFLUOR-positive, ALDEFLUOR-negative). Tumor growth kinetics correlated with the latency and size of tumor formation and the number of ALDEFLUOR-positive cells. No tumor was detected when 500 cells were injected, whereas ALDEFLUOR-positive cells produced tumors that grew at a rate that directly correlated with the number of cells injected. (M) Statistical evaluation of the mammospheres formation efficiency of GI-101A ALDEFLEOR-positive and ALDEFLEOR-negative cells.

The enzyme ALDH1 is a useful marker for isolating healthy human mammary stem cells, progenitor cells as well as transformed tumor-initiating stem cells [12]. The cancer stem-like cells are isolated by virtue of their expression of ALDH1 activity, as assessed by flow cytometry using the ALDEFLUOR assay. To determine whether human breast cancer cell lines contain an ALDEFLUOR-positive population, we analyzed five cell lines of GI-101A, MCF-7, MDA-MB-231, Hs 578 T and SUM149PT, using the ALDEFLUOR assay. In each cell line, we identified an ALDEFLUOR-positive fraction in the size of 6.43 **±** 0.98%, 0.28 **±** 0.06%, 1.74 **±** 0.35%, 1.45 **±** 0.26% and 18.35 **±** 6.54% of the total viable cell population, respectively (Additional file 3: Figure S3). We stained adherent GI-101A cells in plates with ALDEFLUOR present or absent of diethylaminobenzaldehyde (DEAB), an inhibitor of ALDH1 to confirm the flow cytometry results. The fluorescent images indicated the presence of ALDEFLUOR-positive cells in GI-101A and treatment with DEAB reduced ALDH1 activity (Additional file 4: Figure S4). We sorted GI-101A cells for the ALDEFLUOR-positive and ALDEFLUOR-negative populations (Figure 1A-F) by incubating the cells with a fluorescent ALDEFLUOR substrate BODIPY-aminoacetaldehyde (BAAA) and with DEAB, the specific inhibitor of ALDH1 enzyme. The experiment was used to establish the fluorescence baseline of ALDH1- cells and to define the ALDEFLUOR-positive area (ALDH1+) (Figure 1E). We found that incubation of cells with ALDEFLUOR in the absence of DEAB caused a shift in BAAA fluorescence, which defined the ALDEFLUOR-positive cell population (Figure 1D). ALDH1+ and ALDH1- cells were separated under sterile conditions in order to test the tumorigenic potential of GI-101A ALDEFLUOR-positive cells in nude mice mammary fat pad xenografts and in cell culture by mammosphere formation assay. The tumorigenicity of the ALDEFLUOR-positive and ALDEFLUOR-negative cells from GI-101A were tested by inoculating escalating doses of cells (500, 5,000, and 50,000 cells) into mammary fat pads of nude mice. Tumors were detected in mice injected with 50,000 ALDEFLUOR-positive cells as early as five weeks after cell implantation (Figure 1G). ALDEFLUOR-positive cells showed the highest frequency of tumor formation in all times examined (Figure 1H-J). In addition, tumors originating from the ALDEFLUOR-positive cell implantations were much larger than tumors originating from the ALDEFLUOR-negative cells (Figure 1K). The tumor size and latency of tumor formation correlated well with the number of cells injected (Figure 1L). Remarkably, 50,000 and 5,000 ALDEFLUOR-positive cells generated tumors more efficiently than ALDEFLUOR-negative cells. In contrast, we did not see tumors at the 500 cell implantation dose with either ALDEFLUOR-positive or ALDEFLUOR-negative cells. For further characterization, ALDEFLUOR-positive and ALDEFLUOR-negative cells were plated in 96-well ultra-low attachment plates in serum-free medium containing 10 ng/ml EGF and 20 ng/ml bFGF to monitor mammospheres growth under a light microscope. Data obtained indicate that GI-101A ALDEFLUOR-positive cells showed two-fold higher mammosphere formation efficiency as did ALDEFLUOR-negative cells (Figure 1M).

### ALDEFLUOR-positive cells upon culture resemble the parental cell line GI-101A

**Figure 2:**
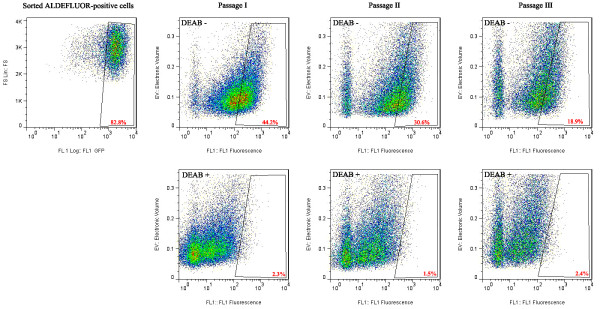
**Expansion and passage of ALDEFLUOR-positive cells results reconstitution of original GI-101A parental cell line.** The sorted ALDEFLUOR-positive cells were seeded in 6-well plates at 5 **×** 10^5^ cells/well and passaged every three days. The cellular outgrowths were re-analyzed in each passage by flow cytometry to assess the reconstitution of parent cell line.

The abilities of self-renewal and differentiation into heterogeneous cell types are the definition of stem cells, which are thought to be functionally mimicked by cancer stem cells [2,29-32]. To assess the capability of cancer stem-like cells to differentiate and reconstitute the composition of a parental cell line, the sorted ALDEFLUOR-positive cells were plated and allowed to expand in cell culture for 12 days. The growing cells were then analyzed for ALDH1 activity by flow cytometry (Figure 2). We found that cultures started with sorted ALDEFLUOR-positive cells resembled the parental cell line after being continuously passaged for three times. In terms of ALDH1 activity, it can be seen that the percentage of ALDEFLUOR-positive cells decreased from 82.8% to 18.9% after three passages in culture. These findings demonstrated that the cancer stem-like cells lose their stem cell marker in cell culture.

### ALDEFLUOR-positive cells exhibited enhanced resistance to chemo- and irradiation treatment when compared to ALDEFLUOR-negative cells

**Figure 3:**
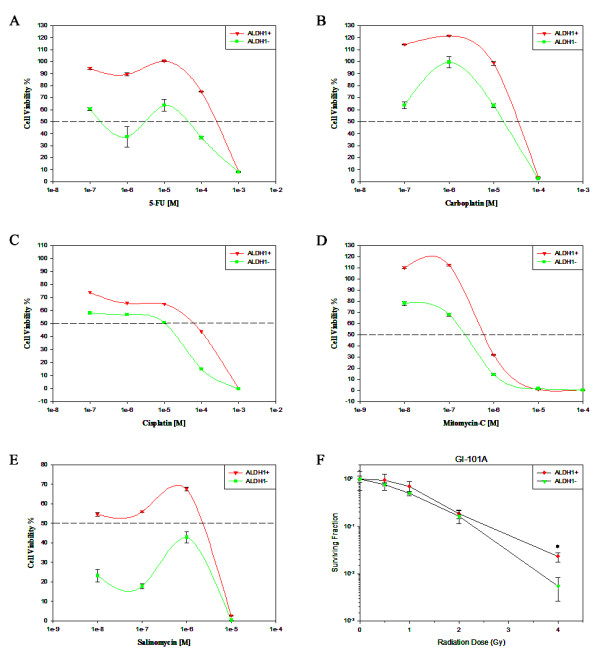
**The response of sorted GI-101A ALDEFLUOR-positive and ALDEFLUOR-negative cells upon chemotherapy and ionizing radiation treatments.** Treatment of GI-101A ALDEFLUOR-positive and ALDEFLUOR-negative cells with (**A**) 5-FU, (**B**) Carboplatin, (**C**) Cisplatin, (**D**) Mitomycin-C and (**E**) Salinomycin or (**F**) ionizing radiation. Means and 95% confidence intervals from three independent experiments are shown (n = 3).

It was reported that cancer stem cells in primary human leukemia and glioblastoma are resistant to chemotherapy [33,34]. To document that breast cancer cell line derived cancer stem-like cells are also resistant to chemotherapeutic agents. We treated ALDEFLUOR-positive and ALDEFLUOR-negative cells with increasing doses of breast cancer drugs such as 5-FU (10^-7^, 10^-6^, 10^-5^, 10^-4^, 10^-3^ mol/L), Carboplatin (10^-7^, 10^-6^, 10^-5^, 10^-4^, mol/L), Cisplatin (10^-7^, 10^-6^, 10^-5^, 10^-4^, 10^-3^ mol/L), Mitomycin-C (10^-8^, 10^-7^, 10^-6^, 10^-5^, 10^-4^ mol/L) and Salinomycin (10^-7^, 10^-6^, 10^-5^, 10^-4^, 10^-3^ mol/L). ALDEFLUOR-positive cells exhibited a significantly higher survival rate in the presence of drugs than the ALDEFLUOR-negative cells (Figure 3A-E). Furthermore, we investigated the sensitivity of the two separated fractions of GI-101A breast cancer cells to irradiation. Cell survival assays were performed using increased doses from 0 Gy, 0.5 Gy, 1 Gy, 2 Gy and 4 Gy. We found that ALDEFLUOR-positive cells showed an increased survival after irradiation treatment in contrast to ALDEFLUOR-negative cell fraction, which exhibited less resistance to radiation treatment (Figure 3F).

### ALDEFLUOR-positive cells showed higher expression levels of CD44, CD49f and CD24 surface marker proteins than ALDEFLUOR-negative cells

**Figure 4:**
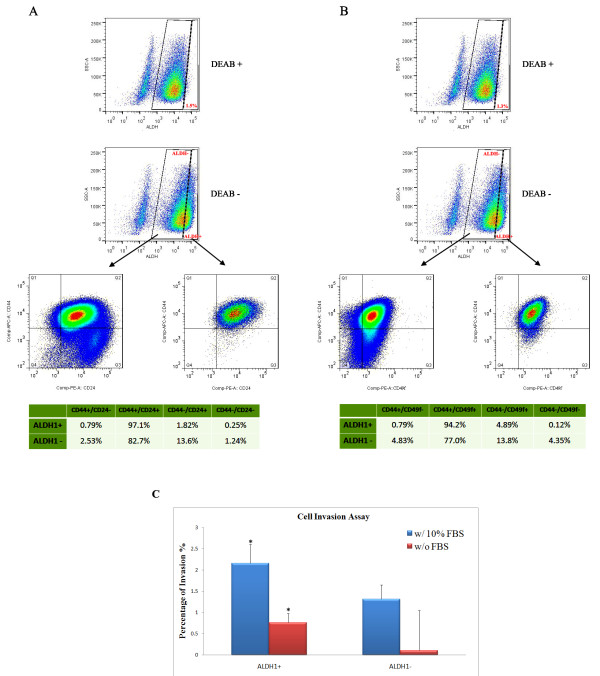
**Metastatic potential of GI-101A ALDEFLUOR-positive and ALDEFLUOR-negative cells.** ALDEFLUOR assay based flow cytometry analysis of human breast cancer stem cell surface markers (**A**) CD44/CD24 and (**B**) CD44/CD49f percentage of each population was summarized in table. (**C**) The migration and invasion ability of ALDEFLUOR-positive and ALDEFLUOR-negative cells. Bars denote the standard error mean (n = 3). The “*” indicate the significance between ALDEFLOUR-positive and ALDEFLUOR-negative cells.

In breast tumors, a CD44^+^CD24^–/low^ESA^+^ lineage subpopulation was originally identified as the tumorigenic (tumor-initiating) fraction. This conclusion is based more on the ability of these cells to form tumors in NOD/SCID mice when injected at very low numbers [6]. Human breast cancer cell lines contain CD44^+^CD24^–/low^ESA^+^ cells that exhibit characteristic stem cell features like anchorage-independent growth at clonal densities, the ability to reconstruct the parental cell fractions, along with enhanced tumorigenicity in mice [9,31]. The CD44^+^CD24^–/low^ phenotype also correlates to the enhanced expression of pro-invasive genes such as IL-1α, IL-6, IL-8, urokinase plasminogen activator [UPA] and the ability to form distant metastases [35-37]. In addition, tumorigenicity of prospective breast CSCs has been linked to the expression of α6 integrin (CD49f) [38] and β1 integrin [39]. To analyze the CD44/CD24/CD49f expression in ALDEFLUOR-positive and ALDEFLUOR-negative cells, we performed an ALDEFLUOR assay followed by a CD44, CD24 and CD49f immunostaining of ALDEFLUOR-positive and ALDEFLUOR-negative cells (Figure 4). As shown in Figure 4A-B, the percentage of CD44^+^ in ALDEFLUOR-positive cells reached to 97.89% (Figure 4A gate Q1 + Q2) or 94.99% (Figure 4B gate Q1 + Q2). However, the percentage of CD44^+^ in ALDEFLUOR-negative cells dropped to 85.23% or 81.83%. Similarly, the percentage of CD49f^+^ in ALDEFLUOR-positive cells reached 99.09% (Figure 4B gate Q2 + Q3) and the percentage of CD49f^+^ in ALDEFLUOR-negative cells dropped to 90.8%. Furthermore, there was no significant difference in the CD24 expression between ALDEFLUOR-positive cells (98.92%, Figure 4A gate Q2 + Q3) and ALDEFLUOR-negative cells (96.3%). Considering those surface marker expression in combination, the percentage of CD44^+^CD24^-^ in ALDEFLUOR-positive cells (0.79%, Figure 4A gate Q1) was much lower than that in ALDEFLUOR-negative cells (2.53%, Figure 4A gate Q1). And the percentage of CD44^+^CD49f^+^ in ALDEFLUOR-positive cells (94.2%, Figure 4B gate Q2) was higher than that in ALDEFLUOR-negative cells (77.0%, Figure 4B gate Q2). Considering both CD44 and CD49f could contribute to carcinoma progression and cancer metastases [40,41] and the ALDEFLUOR-positive breast cancer cells have also been reported to have cell invasion potential *in vitro*, which is related to metastases *in vivo*[42,43], we also performed Basement Membrane Extract (BME) invasion assay, using 10% fetal bovine serum as attractant, to examine the ability of ALDEFLUOR-positive and ALDEFLUOR-negative cells from GI-101A to invade in matrix gels. As shown in Figure 4C, we found that ALDEFLUOR-positive cells demonstrated higher migration capability through BME than the ALDEFLUOR-negative cells. These results supported the notion that GI-101A ALDEFLUOR-positive cells exhibited invasive behavior.

### VACV GLV-1h68 strain replicates more efficiently in ALDEFLUOR-positive cells than in ALDEFLUOR-negative cells in culture

**Figure 5:**
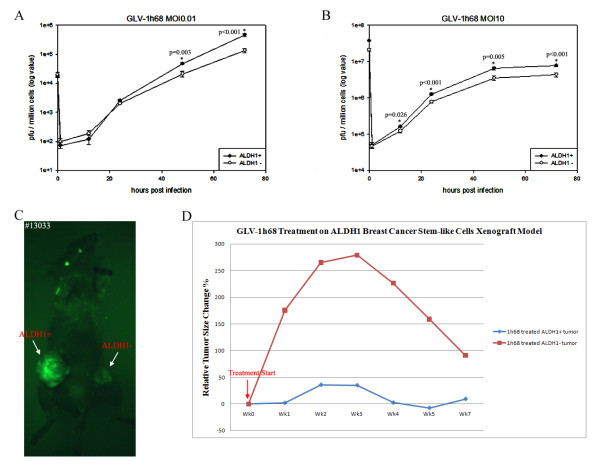
**GLV-1h68 strain preferentially replicates in ALDEFLUOR-positive cell population of GI-101A and enhanced replication in the tumors derived from ALDEFLUOR-positive cells.** Preferential replication of GLV-1h68 strain at (**A**) MOI0.01 and (**B**) MOI10 in ALDEFLUOR-positive cell population of GI-101A breast cancer cells followed by viral titration in CV-1 cells. (**C**) Enhanced fluorescent GFP protein expression in ALDEFLUOR-positive cells derived tumor in comparison to ALDEFLUOR-negative cells. (**D**) Enhanced inhibition of tumor growth in ALDEFLUOR-positive cells derived tumors in comparison to ALDEFLUOR-negative cells derived tumors.

We compared replication efficiency of the vaccinia virus strain GLV-1h68 in sorted ALDEFLUOR-positive and ALDEFLUOR-negative cells. Both cell cultures were infected with GLV-1h68 at an MOI of 0.01 or 10, and the viral titer was determined at 1, 12, 24, 48 and 72 hours post infection. We found that at 72 hours post infection, the viral titer in ALDEFLUOR-positive cells was three times higher at MOI of 0.01, and two times higher at MOI of 10 when compared to ALDEFLUOR-negative cells (Figure 5 A-B).

### VACV GLV-1h68 strain eradicates tumor xenografts derived from ALDEFLUOR-positive cells more efficiently than that derived from ALDEFLUOR-negative cells

To test the efficacy of oncolytic vaccinia virus GLV-1h68 strain, tumors were established in the mammary fat pads of athymic nude mice by implantation of 5 **×** 10^3^ sorted ALDEFLUOR-positive and ALDEFLUOR-negative GI-101A cells. To facilitate the comparison of results, each mouse received two implantations, one into the left and another into the right mammary fat pads, respectively (e.g. 5 **×** 10^3^ ALDEFLUOR-positive cells were placed into right fat pad and 5 **×** 10^3^ ALDEFLUOR-negative cells into left fad pad or vice versa). Twelve weeks after tumor cell implantation, each mouse was administered with 5 **×** 10^6^ pfu of GLV-1h68 retro-orbitally. Both tumor size and virus mediated GFP expression in tumors were monitored weekly. Findings showed that the growth of both tumors was significantly inhibited by virus treatment. However, tumors derived from ALDEFLUOR-positive cells showed a more tumor size reduction after virus treatments, as opposed to tumors derived from ALDEFLUOR-negative implants (Figure 5D). After comparison of the fluorescence images from the tumors, we also found that tumors derived from ALDEFLUOR-positive cells originally had a more efficient GLV-1h68 replication and more fluorescence than tumors derived from ALDEFLUOR-negative cells (Figure 5C). More efficient virus replication resulted in faster tumor regression and earlier fluorescence detection.

### EMT induction of GI-101A cells resulted in cell population with CD44^+^CD24^+^ESA^+^ and CD44^+^CD24^-^ESA^+^ markers

**Figure 6:**
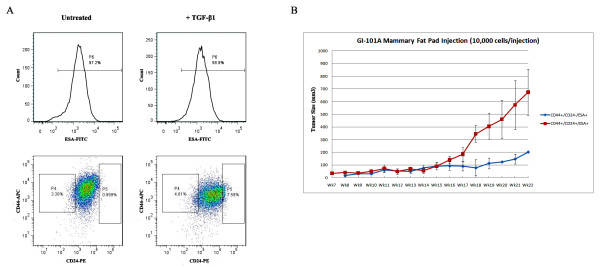
**Separation of tumor-initiating cells from GI-101A upon an EMT induction and the tumorigenicity test.** (**A**) Flow cytometry sorting of tumor-initiating cells from GI-101A upon an EMT induction. CD44^+^CD24^-^ (P4), CD44^+^CD24^+^ (P5) and ESA^+^ (P6) gates were established based on uninduced cells. (**B**) Tumor forming capability of CD44^+^CD24^+^ESA^+^ and CD44^+^CD24^-^ESA^+^ cells were assessed in mice by orthotopic implantation.

We examined whether the treatment of GI-101A cell line with TGF-β1, similar to HMLE cell line [13,14], would lead to an enrichment of mesenchymal cells by losing E-cadherin and enhancing vimentin and fibronectin expression. We found that twelve days after TGF-β1 treatment, EMT was observed in this cell line (Additional file 5: Figure S5). In comparison to GI-101A cells without TGF-β1 treatment, the treated cells showed no difference in CD44 and ESA expression. However, the number of CD24^+^ cells (P5: 7.59%) increased after TGF-β1 treatment, in contrast to cells without TGF-β1 treatment (P5: 0.99%) (Figure 6A). Therefore the treatment of GI-101A cells with TGF-β1 did, in fact, induce a cell population with CD44^+^CD24^+^ESA^+^ marker expression as opposed to CD44^+^CD24^-^ESA^+^. To examine whether CD44^+^CD24^-^ESA^+^ or CD44^+^CD24^+^ESA^+^ cells are cancer stem-like cells and to further investigated their tumorigenic potential, we implanted 10,000, 1,000, and 100 cells into the mammary fat pads of nude mice. Tumors were detected in the mice injected with 10,000 CD44^+^CD24^+^ESA^+^ cells seven weeks after implantation. By 22 weeks after implantation, the tumor size had reached 700 mm^3^ in volume in contrast to 200 mm^3^ of tumors derived from CD44^+^CD24^-^ESA^+^ cells (Figure 6B). Taken together, the CD44^+^CD24^+^ESA^+^ cell population is 3.5 fold more tumorigenic than the CD44^+^CD24^-^ESA^+^ cell population.

VACV GLV-1h68 strain shows higher replication in GI-101A derived CD44^+^CD24^+^ESA^+^ cells resulting in increased eradication of CD44^+^CD24^+^ESA^+^ cells derived tumor xenografts.

**Figure 7:**
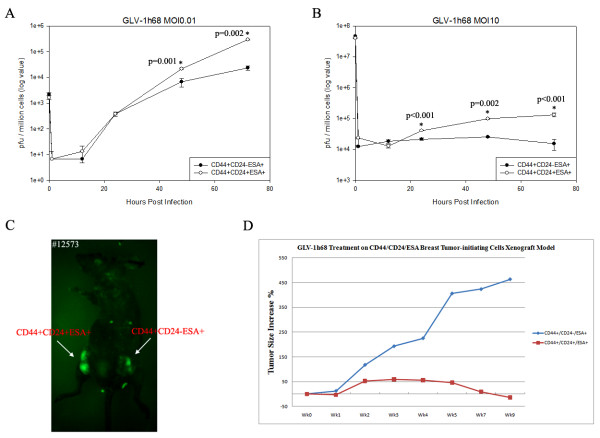
**Enhanced replication of GLV-1h68 strain in CD44**^**+**^**CD24**^**+**^**ESA**^**+**^**cell population of GI-101A and in the tumors derived from CD44**^**+**^**CD24**^**+**^**ESA**^**+**^**cells.** Enhanced replication of GLV-1h68 strain at (**A**) MOI0.01 and (**B**) MOI10 in CD44^+^CD24^+^ESA^+^ cell population of EMT induced GI-101A breast cancer cells followed by viral titration in CV-1 cells. (**C**) Enhanced fluorescent GFP protein expression in CD44^+^CD24^+^ESA^+^ cells derived tumor in comparison to CD44^+^CD24^-^ESA^+^ cells. (**D**) Enhanced inhibition of tumor growth in CD44^+^CD24^+^ESA^+^ derived tumors in comparison to CD44^+^CD24^-^ESA^+^ cells derived tumors.

We tested the ability of two VACVs to replicate in TGF-β1 treated and untreated cells. Twelve days after TGF-β1 treatment of cell lines, HMLE and GI-101A were infected with either GLV-1h68 strain, which carries a GFP fluorescent protein expression cassette, or GLV-1h190 strain, similar to GLV-1h68, but expressing TurboFP635 fluorescent protein. We found a preferential VACV replication resulting in enhanced light emission in the area of EMT cells (Additional file 6: Figure S6). To document higher replication efficiency of GLV-1h68 strain in sorted CD44^+^CD24^+^ESA^+^ in comparison to CD44^+^CD24^-^ESA^+^ cells, we performed a virus infection followed by a replication assay. Both groups of cells were infected with GLV-1h68 at an MOI of 0.01 or 10, and the viral titers were determined at 1, 12, 24, 48 and 72 hours post infection. At 72 hours post infection, the virus titer in CD44^+^CD24^+^ESA^+^ cells was approximately fifteen times higher at MOI 0.01, and ten times higher at MOI 10 in comparison to CD44^+^CD24^-^ESA^+^ cells (Figures 7A-B). To test whether the higher replication efficiency would result in enhanced oncolytic virus efficacy, we established tumors in the mammary fat pads of athymic nude mice by injection of 10,000 of sorted CD44^+^CD24^+^ESA^+^ and CD44^+^CD24^-^ESA^+^ GI-101A cells. In contrast to tumorigenicity experiments described before, each mouse was implanted with both cell fractions in these experiments, one fraction (CD44^+^CD24^+^ESA^+^ ) in right mammary fat pads and the other fraction (CD44^+^CD24^-^ESA^+^) in left, with 10,000 sorted cells. Twenty-two weeks after cell implantation, each mouse received 5 **×** 10^6^ pfu of GLV-1h68 strain via the retro-orbital path. We monitored the tumor size as well as tumor GFP expression weekly by fluorescent imaging (Figure 7C). We found that tumor growth was generally inhibited after virus treatment in both left and right breast tumors. However, tumors derived from CD44^+^CD24^+^ESA^+^ cells showed a greater size reduction than tumors derived from CD44^+^CD24^-^ESA^+^ cells (Figure 7D). In summary, CD44^+^CD24^+^ESA^+^ cells did support a more efficient virus replication and the higher virus titer caused more rapid tumor elimination in nude mice with CD44^+^CD24^+^ESA^+^ cells derived tumor xenografts.

## Discussion

The CSC hypothesis has fundamental implications in cancer biology, in clinical cancer risk assessment, and in the early detection, prognosis, and prevention of cancer. The development of cancer therapeutics based on tumor regression may have produced agents which kill differentiated tumor cells, while a small CSC population become resistant and escape from drug therapy [5]. Cancer stem cell identification was largely based on primary cells as well as early passage of cell lines in a mouse xenograft model [6,44]. However, the success of establishing breast tumor xenografts for cancer stem cell identification has been low mainly due to lack of breast cancer stem cell markers. Cultured and passaged cell lines are readily available to provide more uniform cell populations in mouse xenograft models without the influence of human healthy tissues and human stroma [45,46]. These differentiated cancer cells have a very small number (0.1-1%) of undifferenated cells with cancer stem cell markers [12]. The quantity of cancer stem cells derived from patient samples is limited. Therefore, the establishment of relevant cancer stem-like cell models in cell culture and in mouse tumor xenograft models is critical for the study of cancer stem-like cell characterization and therapy.

According to recently described findings [7], the cell sorter based fluorescent ALDEFLUOR assay, in combination with ALDH1 immunostaining may prove to be critical for the detection and isolation of CSCs from epithelial tumor samples, thus facilitating CSC isolation/identification in the clinical practice and on the cell culture level. In this study, we have successfully identified, isolated and characterized ALDEFLUOR-positive cells from GI-101A breast cancer cell line with cancer stem-like characteristics. We found that less than 5 **×** 10^3^ ALDEFLUOR-positive cells did generate tumors six weeks after implantation in nude mice and did grow to a size of 2,000 mm^3^ at nineteen weeks. Cells with low levels of ALDH1 (ALDEFLUOR-negative) initiated tumor growth at six weeks after implantation in one mouse and did grow remarkably slower, reaching a size of 200 mm^3^ at nineteen weeks. Judging by tumor formation frequency and tumor growth, ALDEFLUOR-positive cell exhibit more cancer stem-like cell growth morphology than ALDEFLUOR-negative cells. These findings were also confirmed by mammosphere formation assay in cell culture. Furthermore, the ALDEFLUOR-positive cells displayed enhanced resistance to five chemotherapy drugs as well as to irradiation in cell cultures. Self-renewal or asymmetrical division is also a unique feature associated with stem cells/cancer stem cells. When the sorted ALDEFLUOR-positive cells were cultured continually on plates, the fraction of ALDEFLUOR-negative cells increased from 17% to 80% approximately. Finally, the ratio between ALDEFLUOR-positive and -negative cells reached to a level, similar to that of GI-101A cell culture before sorting. To further characterize ALDEFLUOR-positive cells, we combined the ALDEFLUOR assay with known breast cancer stem cell surface marker analysis. Interestingly, we found that the ALDEFLUOR-positive cell population exhibited elevated CD44 and CD49f expression compared to the ALDEFLUOR-negative cell population. These findings are consistent with previously published data that both CD44 and CD49f marker proteins contribute to carcinoma progression and cancer metastases [38]. Cancer stem cells may also be responsible for mediating tumor metastases process. A link between cancer stem cells and secondary tumor metastases was first suggested after the identification of “stemness” and an 11-gene signature therapy resistant profile of metastatic and primary tumors in prostate cancer of a transgenic mouse model, as well as in cancer patients [47]. This signature was also a powerful predictor of disease recurrence, survival after therapy, and distant metastases in a variety of cancer types. Here we also demonstrated that ALDEFLUOR-positive cells migrated more efficiently than ALDEFLUOR-negative cells in BME transwells, indicating the increased metastatic potential of GI-101A ALDEFLUOR-positive cells. Contrary to current ‘CD44^+^CD24^-/low^ centric’ thought regarding the developmental plasticity of tumor-initiating mammary epithelial cells [6,48], recent studies have revealed that not only CD44^+^CD24^-/low^ cells can give rise to CD44^+^CD24^+^ cells, as expected for a cancer stem cell [49], but that the converse can also occur; CD44^+^CD24^+^ cells can give rise to their CD44^+^CD24^-/low^ counterparts and single cells from either phenotype are capable of initiating tumors as xenografts with high efficiency [50]. Surprisingly, we also found that ALDEFLUOR-positive cells had enhanced CD24 expression levels. These findings do contradict with known and accepted human breast cancer stem cell markers, which are elevated CD44 and reduced CD24. To confirm that the expression of CD24 is also associated with tumorigenicity, we established a CD44^+^CD24^+^ESA^+^ and a CD44^+^CD24^-^ESA^+^ fraction from GI-101A cell line after EMT induction. In these experiments we showed that CD24^+^ cells initiated tumors more efficiently than the CD24^-^ cells. This explains why ALDEFLUOR-negative cells are less tumorigenic than ALDEFLUOR-positive cells. Since ALDEFLUOR-positive cells showed also higher expression of CD24, similarly to EMT cell population, and both exhibited enhanced tumorigenicity, we suggest that the elevated CD24 expression is a reliable CSC marker in this cell line and that GI-101A cancer stem-like cell markers are ALDH1^+^CD44^+^ CD24^+^ESA^+^. Taken together, our results indicated the phenotype of cancer stem cells is complex and the markers of cancer stem cells vary among different cell sources.

We have reported earlier that after a single intravenous injection of VACV GLV-1h68 strain, more than two dozen human tumors and metastases in nude mice were efficiently colonized and eradicated [51,52]. The importance of cancer stem cells in tumor initiation and their resistance to current therapies require the development of novel therapies for both tumor elimination and prevention of tumor recurrence. The data presented in this manuscript clearly indicate that the VACV GLV-1h68 strain infects the ALDH1^+^CD44^+^CD24^+^ESA^+^ cell population more efficiently than the ALDH1^-^CD44^+^CD24^-^ESA^+^ cell population. The more efficient infection resulted in enhanced cytotoxicity and cell lysis in cell culture. Furthermore, this virus also replicated more efficiently in tumors generated from cancer stem-like cells. Lastly, the enhanced viral replication in the tumor xenograft derived from cancer stem-like ALDH1^+^CD44^+^CD24^+^ESA^+^ cell implantation led to a more efficient elimination of tumors, in comparison to ALDH1^-^CD44^+^CD24^-^ESA^+^ cell derived xenografts. Based on these experiments, either chemotherapy drug resistant or irradiation resistant cancer stem-like cells were identified in cell culture and no further recurrence was seen in treated mice carrying tumors derived from both ALDEFLUOR-positive cell population and CD24^+^ cell population.

## Conclusion

In conclusion, the combination of ALDEFLUOR assay and identification of cancer stem-like cells upon TGF-β1 induced EMT may allow the isolation of cancer stem-like cells from tumor cell lines and tumor biopsies. Further, the cancer stem-like cells showed elevated replication and cytotoxicity of two VACV strains, GLV-1h68 and GLV-1h190. Mice carrying cancer stem-like cell tumor xenografts were efficiently colonized and their tumors were more rapidly eradicated than tumors grown from ALDEFLUOR-negative and CD24^-^ tumor cell population. Therefore, VACV based oncolytic viral therapy may result in efficient eradication of solid tumors, secondary metastases including cancer stem-like cells in tumors as well as circulating tumor cells in blood.

## Abbreviations

CSCs: Cancer stem cells; VACV: Vaccinia virus; ALDH1: Aldehyde dehydrogenase 1; EMT: Epithelial-Mesenchymal Transition; NOD/SCID: Nonobese/severe combined immunodeficient; ATCC: American Type Culture Collection; HMLE: Immortalized human mammary epithelial cells; EGF: Epidermal growth factor; TGF: Transforming growth factor; BME: Basement Membrane Extract; DEAB: Diethylaminobenzaldehyde.

## Competing interests

H. Wang, N. G. Chen, B. R. Minev and A. A. Szalay are salaried employees of Genelux Corporation.

## Authors’ contributions

HW helped with study design, performing and writing of the manuscript. NGC, BRM and AAS conceived the study and study design and guided the writing and editing of the manuscript. All authors read and approved the final manuscript.

## Supplementary Material

Additional file 1 Source, clinical, pathological and molecular features of breast cancer cell lines used in this study. Click here for file

Additional file 2 **Morphology of human breast cancer cell lines in cell culture.** (**A**) GI-101A; (**B**) MCF-7; (**C**) MDA-MB-231; (**D**) Hs 578 T; (**E**) SUM149PT. Click here for file

Additional file 3 Flow cytometry analysis of ALDH activity in human breast cancer cell lines. Click here for file

Additional file 4 **Detection of ALDEFLUOR-positive cells from GI-101A stained by ALDEFLUOR dye *in vitro *with DEAB (C, D) or without DEAB (A, B).** Click here for file

Additional file 5 EMT in HMLE and GI-101A cancer cells: Epithelial marker E-cadherin down-regulated and mesenchymal marker Vimentin and Fibronectin up-regulated. Click here for file

Additional file 6 **Enhanced VACV replication in TGF-β1 treated cells.** (**A**) HMLE and (**B**) GI-101A cells were treated for 12 days followed by infection of GLV-1h68 strain and GLV-1h190 strain at MOI10. The images were taken at 6, 8, 10 and 12 hpi and the red arrows indicate the onset of GFP expression. Click here for file
