# Supplementary material for: Oncolytic vaccinia virus GLV-1h68 strain shows enhanced replication in human breast cancer stem-like cells in comparison to breast cancer cells
Source: J Transl Med. 2012 Aug 17;10:167. doi: 10.1186/1479-5876-10-167 (PMC3478222; doi:10.1186/1479-5876-10-167)
Supplement: Additional file 1 — Source, clinical, pathological and molecular features of breast cancer cell lines used in this study. [file 1479-5876-10-167-S1.pdf]

Table S1. Source, clinical, pathological and molecular features of breast cancer cell lines used in this study.

	Cell line	Gene cluster [1]	ER	PR	HER2	TP53	Source	Tumor type	Age (years)	Ethnicity	Tumorigenicity	Culture media	Culture conditions	Ref
1	GI-101A	Lu	+/-*	-	+	$+^{M}$	XG	IDC	57		Y	RPMI, 20% FBS	37°C, 5% CO_2	[2-4]
2	MCF-7	Lu	+	[-]	Low	$+/-^{WT}$	PE	IDC	69	W	Y**	RPMI, 20% FBS	37°C, 5% CO_2	[5]
3	MDA-MB-231	BaB	-	[-]	-	$++^{M}$	PE	AC	51	W	Y	DMEM, 10% FBS	37°C, 5% CO_2	[6]
4	Hs 578T	BaB	-	[-]		$+^{M}$	P.Br	IDC	74	W	N	RPMI, 20% FBS	37°C, 5% CO_2	[7]
5	SUM149PT	BaB	[-]	[-]	-	[+]	P.Br	Inf Duc.Ca***			Y	Ham's F12, 5% FBS-IH	37°C, 5% CO_2	[8]

AC, adenocarcinoma; BaB, Basal B; Duc.Ca, ductal carcinoma; IDC, invasive ductal carcinoma; Inf, inflammatory; Lu, luminal; P.Br, primary breast; PE, pleural effusion; W, White. ER/PR/HER2/TP53 status: ER/PR positivity, HER2 overexpression, and TP53 protein levels and mutational status (obtained from the Sanger web site; M, mutant protein; WT, wild-type protein) are indicated; XG, xenograft.

Square brackets indicate that levels are inferred from mRNA levels alone where protein data is not available.

Media conditions: FBS, fetal bovine serum; I, Insulin (5µg/ml); H, hydrocortisone (1µg/ml); DMEM, Dulbecco's modified Eagle's medium; RPMI, RPMI medium 1640; Ham's F12, F-12 nutrient mixture (Ham).

* Positive in mRNA levels and negative in protein level.

**With estrogen supplement.

***Information obtained on Asterand website (http://solutions.asterand.com/Human-Cell-Lines-s/1.htm).

1. Neve RM, Chin K, Fridlyand J, Yeh J, Baehner FL, Fevr T, Clark L, Bayani N, Coppe JP, Tong F, et al: **A collection of breast cancer cell lines for the study of functionally distinct cancer subtypes.** *Cancer Cell* 2006, **10:**515-527.
2. Rathinavelu P, Malave A, Raney SR, Hurst J, Roberson CT, Rathinavelu A: **Expression of mdm-2 oncoprotein in the primary and metastatic sites of mammary tumor (GI-101) implanted athymic nude mice.** *Cancer Biochem Biophys* 1999, **17:**133-146.
3. Morrissey JJ, Raney S: **A metastatic breast tumor cell line, GI-101A, is estrogen receptor positive and responsive to estrogen but resistant to tamoxifen.** *Cell Biol Int* 1998, **22:**413-419.
4. Hurst J, Maniar N, Tombarkiewicz J, Lucas F, Roberson C, Steplewski Z, James W, Perras J: **A novel model of a metastatic human breast tumour xenograft line.** *Br J Cancer* 1993, **68:**274-276.
5. Osborne CK, Hobbs K, Trent JM: **Biological differences among MCF-7 human breast cancer cell lines from different laboratories.** *Breast Cancer Res Treat* 1987, **9:**111-121.
6. Cailleau R, Olive M, Cruciger QV: **Long-term human breast carcinoma cell lines of metastatic origin: preliminary characterization.** *In Vitro* 1978, **14:**911-915.
7. Lacroix M, Leclercq G: **Relevance of breast cancer cell lines as models for breast tumours: an update.** *Breast Cancer Res Treat* 2004, **83:**249-289.
8. van Golen KL, Wu ZF, Qiao XT, Bao LW, Merajver SD: **RhoC GTPase, a novel transforming oncogene for human mammary epithelial cells that partially recapitulates the inflammatory breast cancer phenotype.** *Cancer Res* 2000, **60:**5832-5838.
