# Supplementary figures and images for: Oncolytic vaccinia virus GLV-1h68 strain shows enhanced replication in human breast cancer stem-like cells in comparison to breast cancer cells
Source: J Transl Med. 2012 Aug 17;10:167. doi: 10.1186/1479-5876-10-167 (PMC3478222; doi:10.1186/1479-5876-10-167)

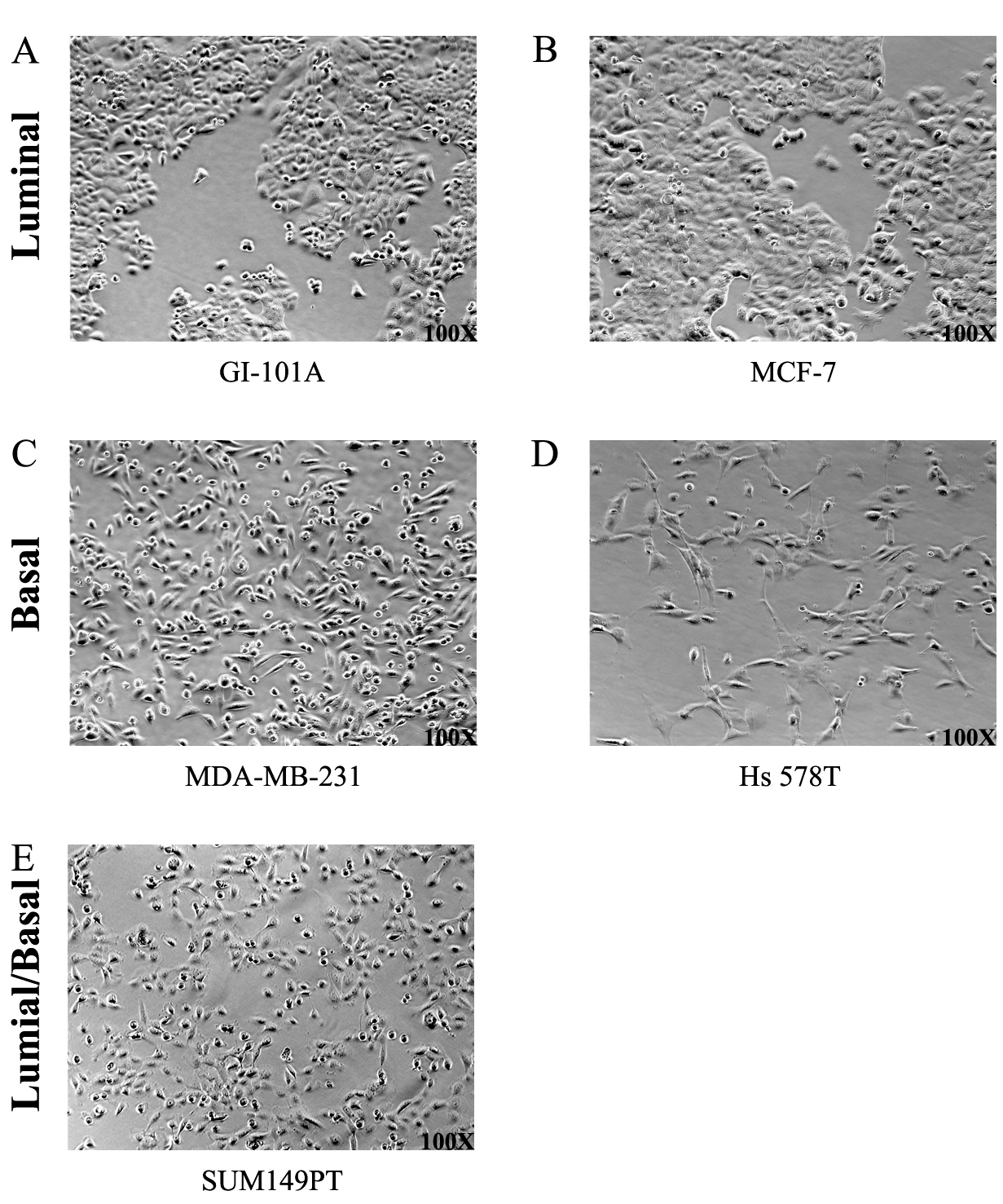

Supplement: Additional file 2 — Morphology of human breast cancer cell lines in cell culture. (A) GI-101A; (B) MCF-7; (C) MDA-MB-231; (D) Hs 578 T; (E) SUM149PT. [file 1479-5876-10-167-S2.tiff]

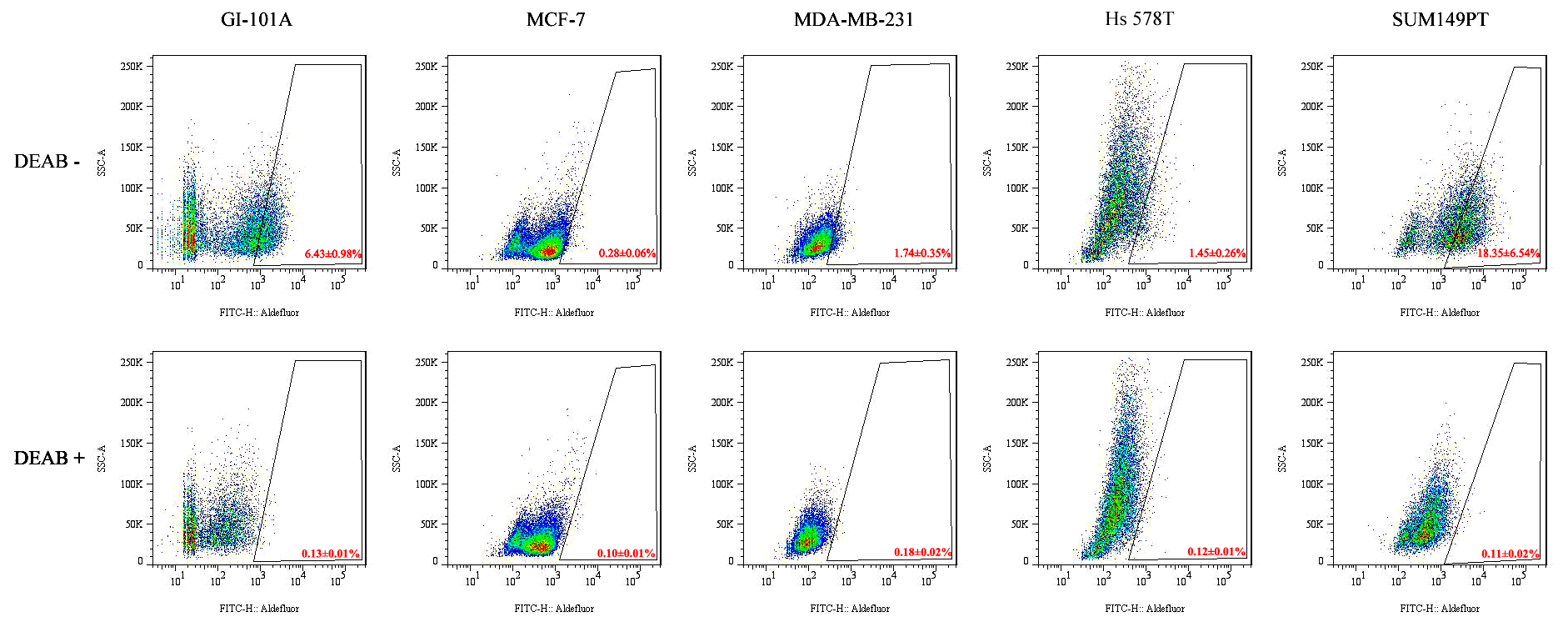

Supplement: Additional file 3 — Flow cytometry analysis of ALDH activity in human breast cancer cell lines. [file 1479-5876-10-167-S3.tiff]

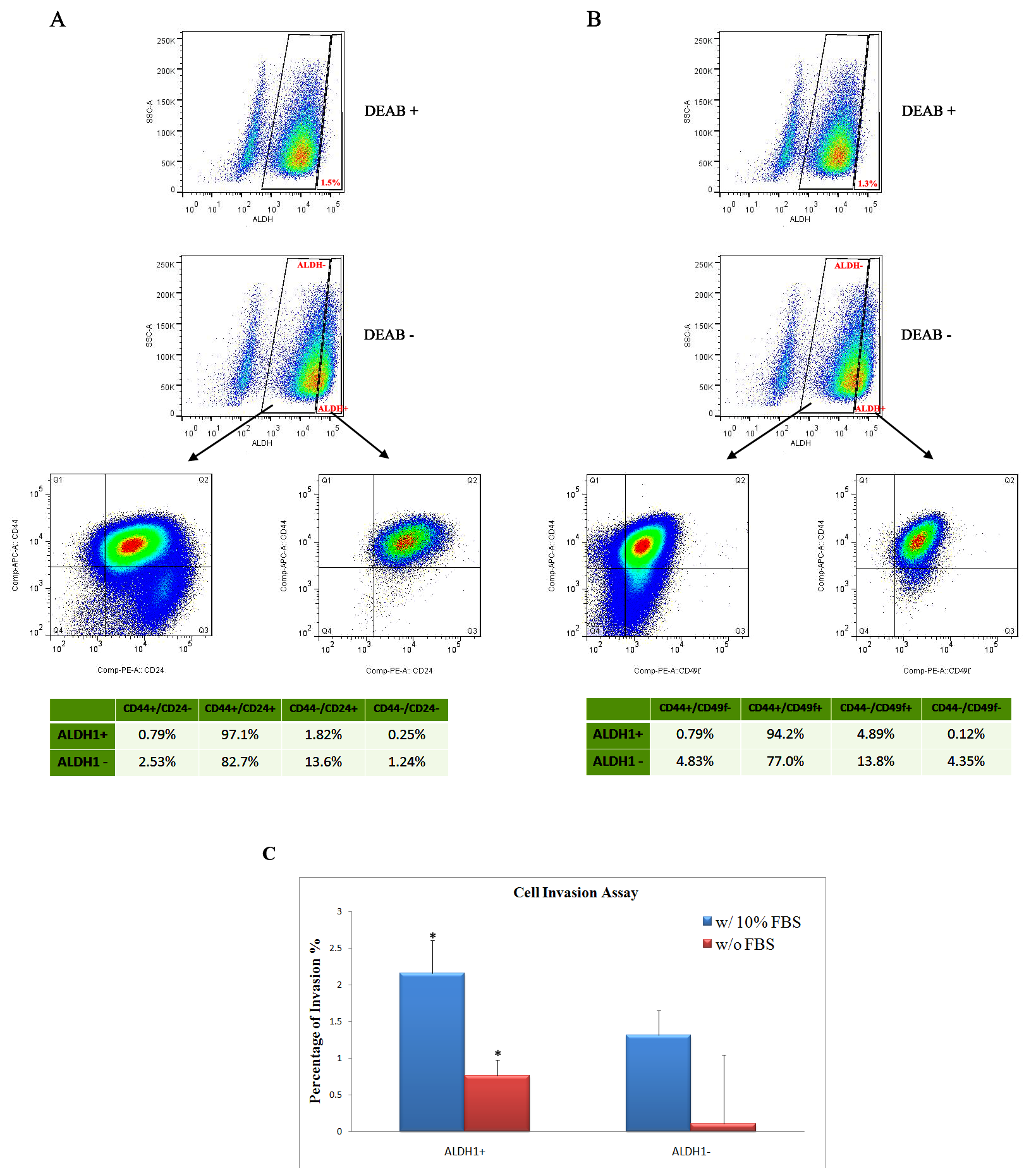

Supplement: Additional file 4 — Detection of ALDEFLUOR-positive cells from GI-101A stained by ALDEFLUOR dye in vitro with DEAB (C, D) or without DEAB (A, B). [file 1479-5876-10-167-S4.tiff]

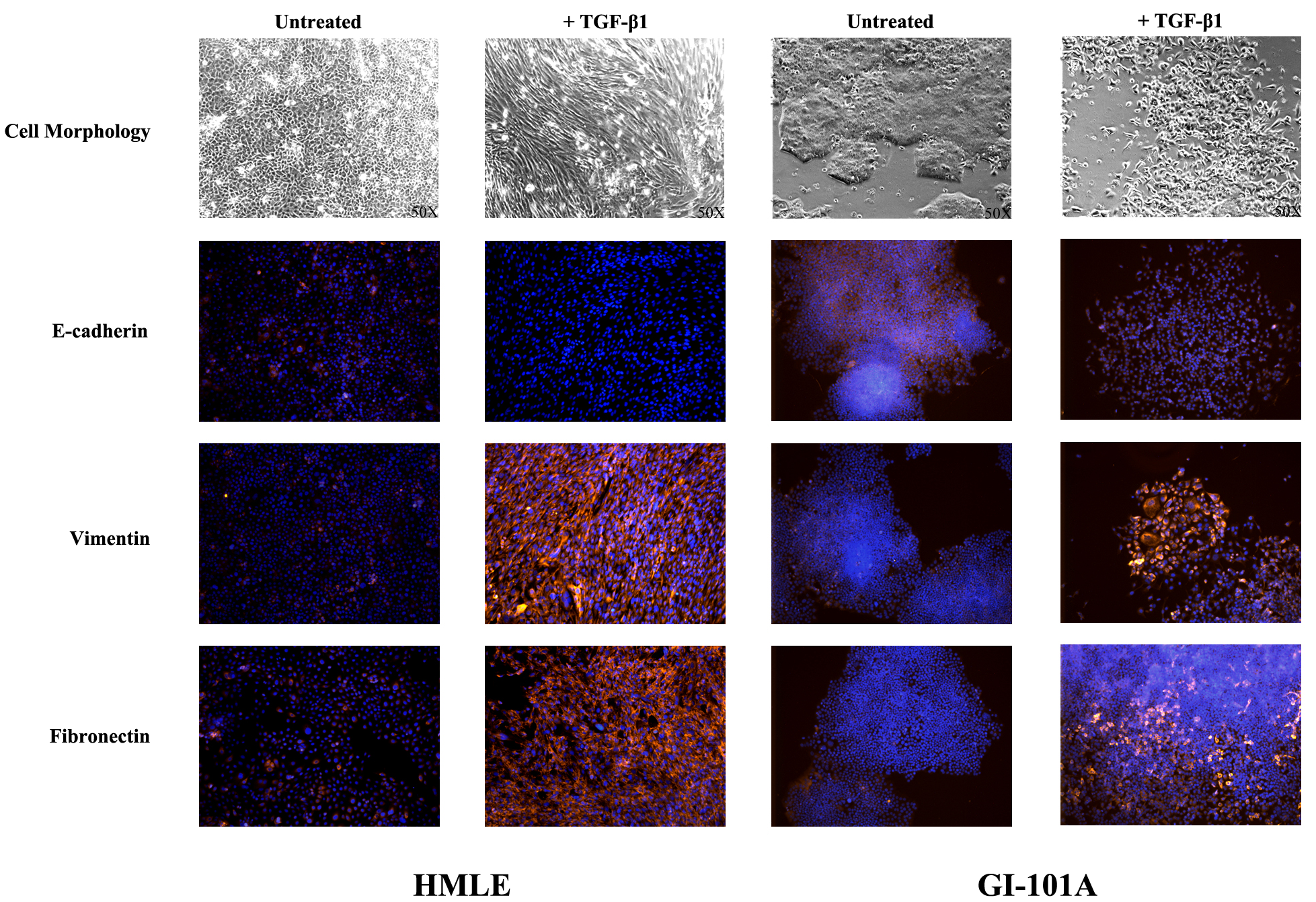

Supplement: Additional file 5 — EMT in HMLE and GI-101A cancer cells: Epithelial marker E-cadherin down-regulated and mesenchymal marker Vimentin and Fibronectin up-regulated. [file 1479-5876-10-167-S5.tiff]

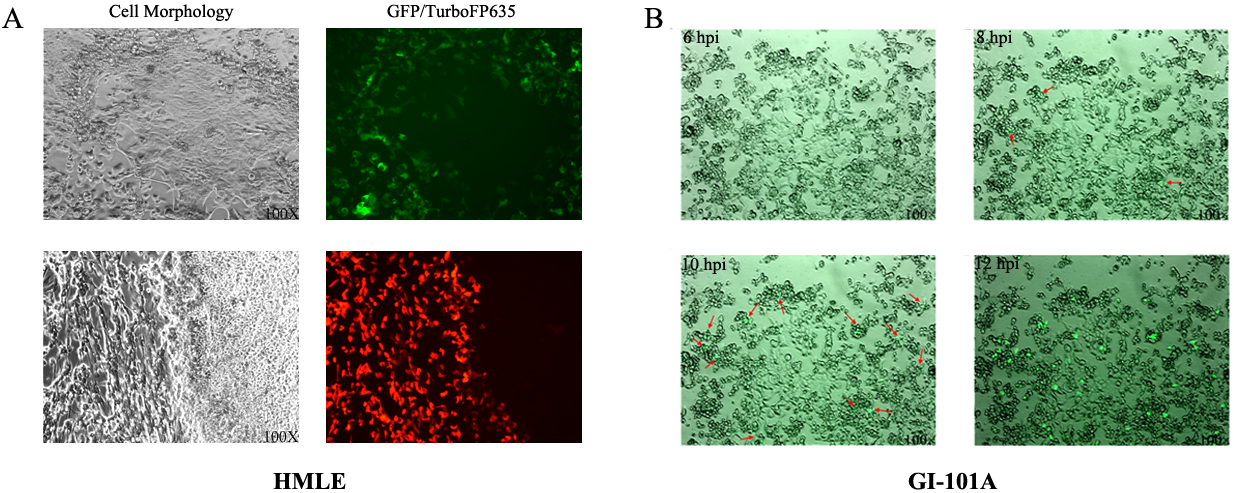

Supplement: Additional file 6 — Enhanced VACV replication in TGF-β1 treated cells. (A) HMLE and (B) GI-101A cells were treated for 12 days followed by infection of GLV-1h68 strain and GLV-1h190 strain at MOI10. The images were taken at 6, 8, 10 and 12 hpi and the red arrows indicate the onset of GFP expression. [file 1479-5876-10-167-S6.tiff]
